# Protodioscin Restores Ovarian Function and Suppresses p53–Bax/Bcl-2–Mediated Apoptosis in a Doxorubicin-Induced Model of Ovarian Toxicity

**DOI:** 10.34172/apb.46593

**Published:** 2026-07-11

**Authors:** Amoura M. Abou-El-Naga, Aya M. Abd-Elhady, Mohamed E. Abdraboh, Yasmin M. Tag, Maher Monir. Akl

**Affiliations:** ^1^Department of Zoology, Faculty of Science, Mansoura University, 35516, Mansoura, Egypt; ^2^Faculty of Oral and Dental Medicine, Delta University for Science and Technology, Gamasa, 35516, Egypt; ^3^Faculty of Medicine, National Research Lobachevsky State University of Nizhny Novgorod, 603022, Nizhny Novgorod, Russia

**Keywords:** Protodioscin, Doxorubicin, Ovarian toxicity, Apoptosis, Bax/Bcl-2, Gonadotropins

## Abstract

**Purpose:**

This study evaluated the protective effects of protodioscin against doxorubicin (DOX)-induced ovarian injury by examining its potential to modulate hormonal alterations and apoptosis-related pathways in a rat model.

**Methods:**

Forty female Wistar rats were randomized into four groups: control, protodioscin (10 mg/kg orally for 14 days), DOX (10 mg/kg intraperitoneally on Day 7), and DOX plus protodioscin. Serum levels of follicle-stimulating hormone (FSH), luteinizing hormone (LH), anti-Müllerian hormone (AMH), and 17β-estradiol (E2) were quantified using enzyme-linked immunosorbent assay. Ovarian histopathology and immunohistochemistry were performed to evaluate tissue integrity and expression of p53, CDK4, and Survivin. Gene expression of Bax and Bcl-2 was assessed using semi-quantitative reverse transcription polymerase chain reaction (RT-PCR).

**Results:**

DOX administration significantly reduced ovarian weight by 38.5% and uterine weight by 22.6% (*P*<0.01) compared with controls, accompanied by marked hormonal alterations, including increased FSH and decreased LH, AMH, and E2 levels. DOX also increased p53 and Bax expression and decreased Bcl-2 expression, resulting in an elevated Bax/Bcl-2 ratio. Co-treatment with protodioscin significantly attenuated these changes, partially restoring hormonal levels and modulating apoptosis-related markers. Protodioscin reduced p53 and Bax expression while increasing Bcl-2, CDK4, and Survivin levels, leading to a marked reduction in the Bax/Bcl-2 ratio (*P*<0.05).

**Conclusion:**

Protodioscin exerts a protective effect against DOX-induced ovarian injury, likely through modulation of hormonal disturbances and apoptosis-related pathways. However, these findings should be interpreted with caution due to the absence of estrous cycle synchronization and the use of an acute DOX model.

## Introduction

 Doxorubicin (DOX), a potent anthracycline chemotherapeutic agent, is widely employed in treating various malignancies, including breast cancer, lymphomas, and leukemias.^1^ Despite its efficacy, its clinical application is hindered by significant cytotoxicity to non-target organs, with the ovary being particularly vulnerable.^2^ In premenopausal women, DOX-induced ovarian damage may lead to premature ovarian insufficiency, characterized by follicular atresia, depletion of the ovarian reserve, reduced estrogen levels (hypoestrogenism), and infertility.^3,4^ At the molecular level, DOX exerts its antineoplastic effects by intercalating into DNA and stabilizing the topoisomerase II–DNA cleavage complex, leading to double-stranded DNA breaks and replication arrest.^5^ These genotoxic events activate the p53 signaling pathway, a key regulator of the DNA damage response. Activated p53 upregulates pro-apoptotic genes such as *Bax* while downregulating anti-apoptotic factors like *Bcl-2*, promoting mitochondrial outer membrane permeabilization, cytochrome c release, and caspase activation.^6,7^ This intrinsic apoptotic cascade results in the loss of granulosa cells and oocytes, which are essential for folliculogenesis and steroidogenesis.^8^ Concurrently, DOX-induced oxidative stress, driven by excessive reactive oxygen species (ROS) production in mitochondria, exacerbates DNA fragmentation and lipid peroxidation, further compromising follicular integrity and vascular function.^9^ These alterations are associated with decreased circulating levels of anti-Müllerian hormone (AMH) and 17β-estradiol (E2), alongside a compensatory rise in follicle-stimulating hormone (FSH), reflecting disruption of ovarian endocrine function. However, since hormonal levels are influenced by the estrous cycle stage, interpretation of endocrine findings requires cautious consideration in experimental settings. Despite advances in fertility preservation during chemotherapy, including gonadotropin-releasing hormone (GnRH) analogs and cryopreservation strategies, these approaches remain limited by variable efficacy and technical challenges.^10,11^

 Therefore, there is a critical need for pharmacological agents capable of protecting ovarian function without compromising the anticancer efficacy of chemotherapy. In this context, steroidal saponins, particularly protodioscin a natural furostanol saponin derived from plants such as *Tribulus terrestris* have demonstrated promising biological effects in experimental models.^12^ Protodioscin exhibits structural similarity to endogenous steroid hormones, which may enable modulation of gonadotropin signaling and enhancement of ovarian responsiveness. Experimental evidence suggests that protodioscin can support steroidogenesis and partially improve endocrine balance under stress conditions.^13^ Furthermore, protodioscin has been reported to exert antioxidant and anti-apoptotic effects, potentially through modulation of the p53–Bax/Bcl-2 axis and preservation of mitochondrial integrity.^14^ However, whether these effects are mediated through direct ovarian actions or secondary to attenuation of systemic DOX toxicity remains unclear. Additionally, conclusions regarding central hypothalamic–pituitary–gonadal (HPG) axis regulation cannot be definitively established based solely on peripheral measurements. Accordingly, the present study aimed to evaluate the potential protective effects of protodioscin against DOX-induced ovarian injury in a rat model. The study focused on biochemical, histopathological, and molecular parameters, with particular emphasis on apoptosis-related pathways and hormonal alterations.

## Materials and Methods

###  Chemicals

 Protodioscin (≥ 98% purity) was procured from Sigma-Aldrich (St. Louis, MO, USA) and stored at −20 °C in a dry, dark environment until use. For oral administration, the compound was freshly suspended in 0.5% carboxymethylcellulose (CMC) and delivered via gastric gavage at a dose of 10 mg/kg body weight daily for 14 days. The selected dose was based on previously reported studies demonstrating its efficacy in improving reproductive function without detectable adverse effects. Doxorubicin hydrochloride (DOX) injection (50 mg/25 mL; Pfizer Inc., USA) was administered as a single intraperitoneal dose of 10 mg/kg on Day 7 to induce ovarian injury. This dosing regimen was selected based on previously published experimental models of chemotherapy-induced ovarian toxicity; however, it is acknowledged that this approach may reflect acute toxicity rather than a fully chronic clinical model of premature ovarian insufficiency. All other chemicals, reagents, and assay kits used in biochemical, histological, and molecular analyses were of analytical grade and obtained from standard commercial suppliers.

###  Experimental Animals

 Forty healthy adult female Wistar rats (2–3 months of age, weighing 60–70 g) were obtained from the animal facility of the Institute of Serum and Vaccine Production, Cairo University. Animals were housed in opaque polypropylene cages (56 × 30 × 28 cm) under controlled environmental conditions (temperature 24 ± 1 °C, relative humidity 55 ± 5%, 14 h light/10 h dark cycle) with free access to a standard pelleted diet and water ad libitum.

 Animals were acclimatized for one week prior to the experiment. During this period, any abnormal or unhealthy animals were excluded. Cages were routinely cleaned and disinfected to maintain hygienic conditions and minimize stress-related variability. All experimental procedures were conducted in accordance with the guidelines of the Bioethics Committee of Mansoura University.

###  Experimental Groups

 Rats were randomly assigned into four experimental groups (n = 10 per group) to evaluate the protective effects of protodioscin against DOX-induced ovarian injury. The experimental design is summarized in Table 1.

**Table 1:** Experimental Grouping and Treatment Schedule

**Group**	**Treatment Description**	**Doxorubicin (i.p.)**	**Study Purpose**
G1 — Control	Vehicle (0.5% CMC) orally for 14 days	—	Negative control
G2 — Protodioscin	Protodioscin 10 mg/kg orally for 14 days	—	Compound control
G3 — DOX	Vehicle orally for 14 days	10 mg/kg (Day 7)	Toxicity model
G4 — DOX + Protodioscin	Protodioscin 10 mg/kg orally for 14 days	10 mg/kg (Day 7)	Protective group

 Protodioscin administration commenced on Day 1 and continued daily for 14 days, while DOX was administered once on Day 7. Animals were monitored daily for general behavior, food and water intake, and body weight, which was recorded on Days 0, 7, and 14. The primary outcome of the study was defined as preservation of ovarian structure and endocrine function, assessed by ovarian histology and serum levels of AMH and E2. Secondary outcomes included gonadotropin levels (FSH, LH), immunohistochemical markers (p53, CDK4, Survivin), and gene expression of apoptosis-related markers (*Bax* and *Bcl-2*).

###  Sample Collection and Tissue Preparation

 At the end of the experimental period (Day 15), animals were anesthetized using diethyl ether, and blood samples were collected via cardiac puncture into non-heparinized tubes. Serum was separated by centrifugation at 3000 rpm for 15 minutes and stored at −20 °C until analysis. All animals were sacrificed at a consistent time point to minimize circadian variation. However, estrous cycle staging was not monitored or synchronized prior to sampling, which is recognized as a limitation when interpreting hormonal and histological findings.

 Following blood collection, the abdominal cavity was opened, and ovaries and uteri were carefully excised, cleared of surrounding tissues, blotted dry, and weighed. One ovary from each animal was fixed in 10% neutral-buffered formalin for histopathological and immunohistochemical analysis, while the contralateral ovary was snap-frozen in liquid nitrogen and stored at −80 °C for gene expression analysis.

###  Hormonal Detection

 Serum levels of follicle-stimulating hormone (FSH), luteinizing hormone (LH), anti-Müllerian hormone (AMH), and 17β-estradiol (E2) were measured using commercially available enzyme-linked immunosorbent assay (ELISA) kits (CUSABIO, Wuhan, China), according to the manufacturer’s instructions. The catalog numbers were as follows: FSH (CSB-E06869r), LH (CSB-E12654r), AMH (CSB-E11162r), and E2 (DEIA-S22). All samples were analyzed in duplicate, and absorbance was measured at 450 nm using a microplate reader. Hormone concentrations were calculated from standard calibration curves and expressed in appropriate units.

###  Histopathological and Immunohistochemical Evaluation

 Formalin-fixed ovarian tissues were processed through graded ethanol dehydration, cleared in xylene, and embedded in paraffin wax. Sections (5 µm) were prepared using a rotary microtome and stained with hematoxylin and eosin (H&E) for histopathological examination. Follicular morphology, stromal integrity, vascularization, and degenerative changes were evaluated using light microscopy.

 For immunohistochemistry, 4 µm sections were deparaffinized, rehydrated, and subjected to antigen retrieval using citrate buffer (pH 6.0) at 95 °C for 15 minutes. Endogenous peroxidase activity was blocked using 3% hydrogen peroxide, followed by blocking of nonspecific binding with 5% bovine serum albumin. Sections were incubated overnight at 4 °C with primary antibodies against p53 (Cat. MA5-12453), CDK4 (Cat. AHZ0202), and Survivin (Cat. MA5-15077) (Thermo Fisher Scientific, USA). Appropriate positive and negative controls were included in each staining run. After washing, sections were incubated with biotinylated secondary antibodies, followed by HRP-streptavidin and visualization using 3,3′-diaminobenzidine (DAB).

 For quantitative analysis, three non-overlapping sections per ovary and five random high-power fields per section were analyzed. The animal was considered the true experimental unit, and mean values per animal were calculated to avoid pseudo-replication. Image analysis was performed using ImageJ software by two independent blinded observers.

###  Gene Expression Evaluation for Bax and Bcl-2

 Total RNA was extracted from frozen ovarian tissues using TRIzol reagent (Invitrogen, USA; Cat. No. 15596026), and RNA purity was assessed using A260/A280 ratios. Complementary DNA (cDNA) was synthesized using the RevertAid First Strand cDNA Synthesis Kit (Thermo Fisher Scientific, USA). The primer sequences used for Bax, Bcl-2, and β-actin are presented in Table 2.

**Table 2:** Primer Sequences for Gene-Expression Analysis

**Gene**	**Forward Primer (5′→3′)**	**Reverse Primer (5′→3′)**
Bax	ATCCACCAAGAAGCTGAGCG	TCCACATCAGCAATCATCCTCTG
Bcl-2	GACTGAGTACCTGAACCGGC	AGTTCCACAAAGGCATCCCAG
β-actin	AGAGGGAAATCGTGCGTGAC	CAATAGTGATGACCTGGCCGT

 Gene expression analysis was performed using semi-quantitative reverse transcription PCR (RT-PCR).

 PCR reactions were carried out in a total volume of 25 µL containing PCR Master Mix, primers, cDNA template, and nuclease-free water. Amplified products were separated on agarose gel and visualized under UV illumination. Band intensities were quantified using densitometric analysis, and relative expression levels were normalized to β-actin. It is acknowledged that this method provides relative expression trends rather than precise quantitative measurements.

###  Systemic Safety Assessment

 Serum levels of alanine aminotransferase (ALT), aspartate aminotransferase (AST), and creatinine were measured using colorimetric diagnostic kits (RAM Diagnostics, Cairo, Egypt) according to the manufacturers’ protocols to evaluate potential hepatic and renal toxicity.

###  Statistical Analysis

 Data were analyzed using IBM SPSS Statistics version 21 (Armonk, NY, USA). Results are presented as mean ± standard deviation (SD). Normality was assessed using the Shapiro–Wilk test.

 Intergroup comparisons were performed using one-way analysis of variance (ANOVA) followed by Tukey’s post hoc test for multiple comparisons. In addition, effect sizes (η^2^) were calculated to assess the magnitude of differences between groups. Statistical significance was set at *P* < 0.05. Graphs were generated using GraphPad Prism 9.0 (GraphPad Software, San Diego, CA, USA).

## Results

 The administration of doxorubicin (DOX) induced marked systemic and ovarian toxicity in female Wistar rats, manifested as reductions in body and reproductive organ weights, hormonal imbalances, histopathological alterations in ovarian tissue, elevated pro-apoptotic markers, and dysregulated gene expression favoring cell death.

 Co-treatment with protodioscin (10 mg/kg orally daily for 14 days) significantly mitigated these adverse effects, restoring most parameters toward levels comparable with the control group.

 Data were analyzed using one-way ANOVA followed by Tukey’s post hoc test, with statistical significance set at *P* < 0.05. Results are presented as mean ± SD (n = 10 rats per group).

###  Effects on Body, Ovarian, and Uterine Weights

 DOX administration led to a substantial decrease in body weight, reflecting systemic toxicity, as well as reductions in ovarian and uterine weights, indicative of reproductive organ atrophy. Protodioscin alone slightly increased these weights beyond control levels, suggesting a trophic effect on reproductive tissues. In the co-treatment group, protodioscin significantly attenuated DOX-induced weight loss, indicating a protective effect against chemotherapy-associated systemic and gonadal toxicity (Table 3, Figure 1).

**Table 3:** Effects of protodioscin on body, ovarian, and uterine weights in DOX-treated female rats

**Group**	**Body weight (g)**	**Ovary weight (mg)**	**Uterus weight (mg)**
Control	185.4 ± 5.2	52.8 ± 2.1	248.6 ± 7.3
Protodioscin	192.7 ± 4.9*	56.3 ± 1.8*	255.1 ± 6.5*
DOX	158.9 ± 6.4**	32.5 ± 1.5**	192.4 ± 5.8**
DOX + Protodioscin	179.2 ± 5.7#	48.7 ± 2.0#	239.8 ± 6.9#

Data: mean ± SD. * *P* < 0.05 vs. Control; ** *P* < 0.01 vs. Control; # *P* < 0.05 vs. DOX.

**Figure 1:**
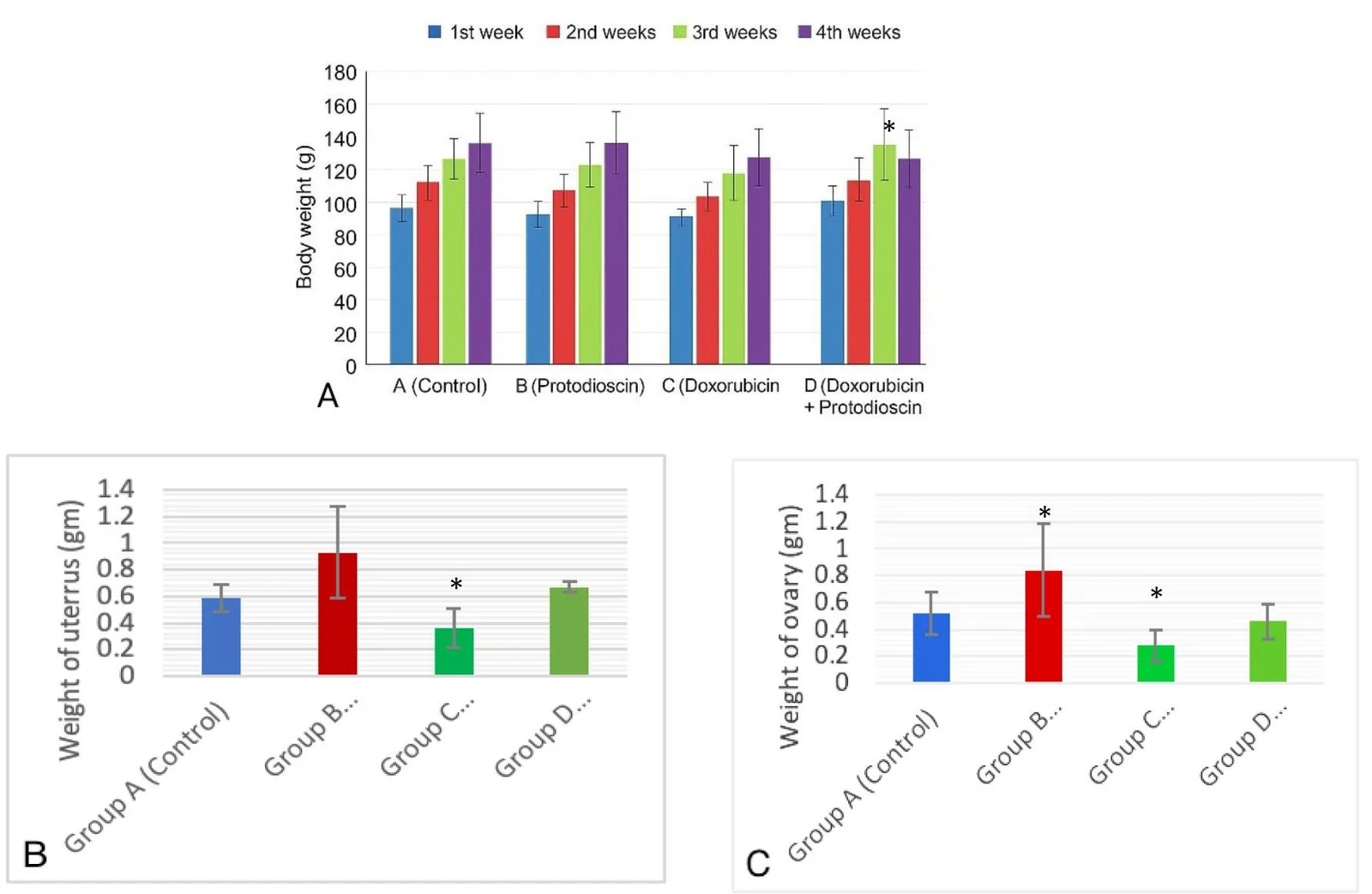
Effects of Protodioscin on Body, Uterine, and Ovarian Weights in DOX-Treated Rats. **A** (Body Weight): Bar chart showing mean body weights (g) across the four experimental groups (Control, Protodioscin 10 mg/kg, DOX 10 mg/kg, and DOX + Protodioscin). Administration of DOX markedly reduced body weight by approximately 14.3% compared with control (*P* < 0.01), indicating systemic cytotoxicity and metabolic suppression. Co-treatment with Protodioscin significantly restored body weight, achieving nearly 12.7% recovery relative to DOX alone (*P* < 0.05). The Protodioscin-only group exhibited a modest increase (+4%) compared to control, suggesting a mild anabolic or trophic metabolic influence. **B** (Ovary Weight): DOX exposure resulted in a 38.4% reduction in ovarian weight versus control (*P* < 0.01), reflecting follicular atresia and stromal degeneration. Protodioscin co-treatment markedly improved ovarian mass by 49.8% relative to the DOX group (*P* < 0.05), while Protodioscin alone caused a mild physiological increase (+6.6%) compared to control. **C** (Uterus Weight): DOX induced a 22.6% decline in uterine weight (*P* < 0.01), consistent with gonadal atrophy and estrogen deficiency. Co-administration of Protodioscin restored uterine mass by 24.6% (*P* < 0.05), approaching control levels and indicating recovery of hormonal function. Data are presented as mean ± SD; one-way ANOVA followed by LSD post hoc test (n = 10 rats/group)

###  Effects on Serum Reproductive Hormones

 DOX administration was associated with significant hormonal alterations, including increased FSH levels and decreased LH, AMH, and E2 levels, consistent with impaired ovarian endocrine function. However, given that hormonal measurements are influenced by the estrous cycle stage, these findings should be interpreted with caution. Protodioscin co-treatment significantly attenuated these alterations, with reductions in FSH and increases in LH, AMH, and E2 compared with the DOX group, suggesting partial restoration of hormonal balance (Table 4; Figure 2).

**Table 4:** Serum levels of reproductive hormones in experimental groups

**Group**	**FSH (mIU/mL)**	**LH (mIU/mL)**	**AMH (ng/mL)**	**E2 (pg/mL)**
Control	5.3 ± 0.4	6.1 ± 0.3	4.5 ± 0.2	47.2 ± 2.6
Protodioscin	5.1 ± 0.3	6.4 ± 0.2*	4.8 ± 0.2*	51.3 ± 2.4*
DOX	9.2 ± 0.5**	3.2 ± 0.2**	2.4 ± 0.1**	29.1 ± 1.9**
DOX + Protodioscin	5.7 ± 0.4#	5.8 ± 0.3#	4.2 ± 0.2#	44.9 ± 2.1#

Data: mean ± SD. * *P* < 0.05 vs. Control; ** *P* < 0.01 vs. Control; # *P* < 0.05 vs. DOX.

**Figure 2:**
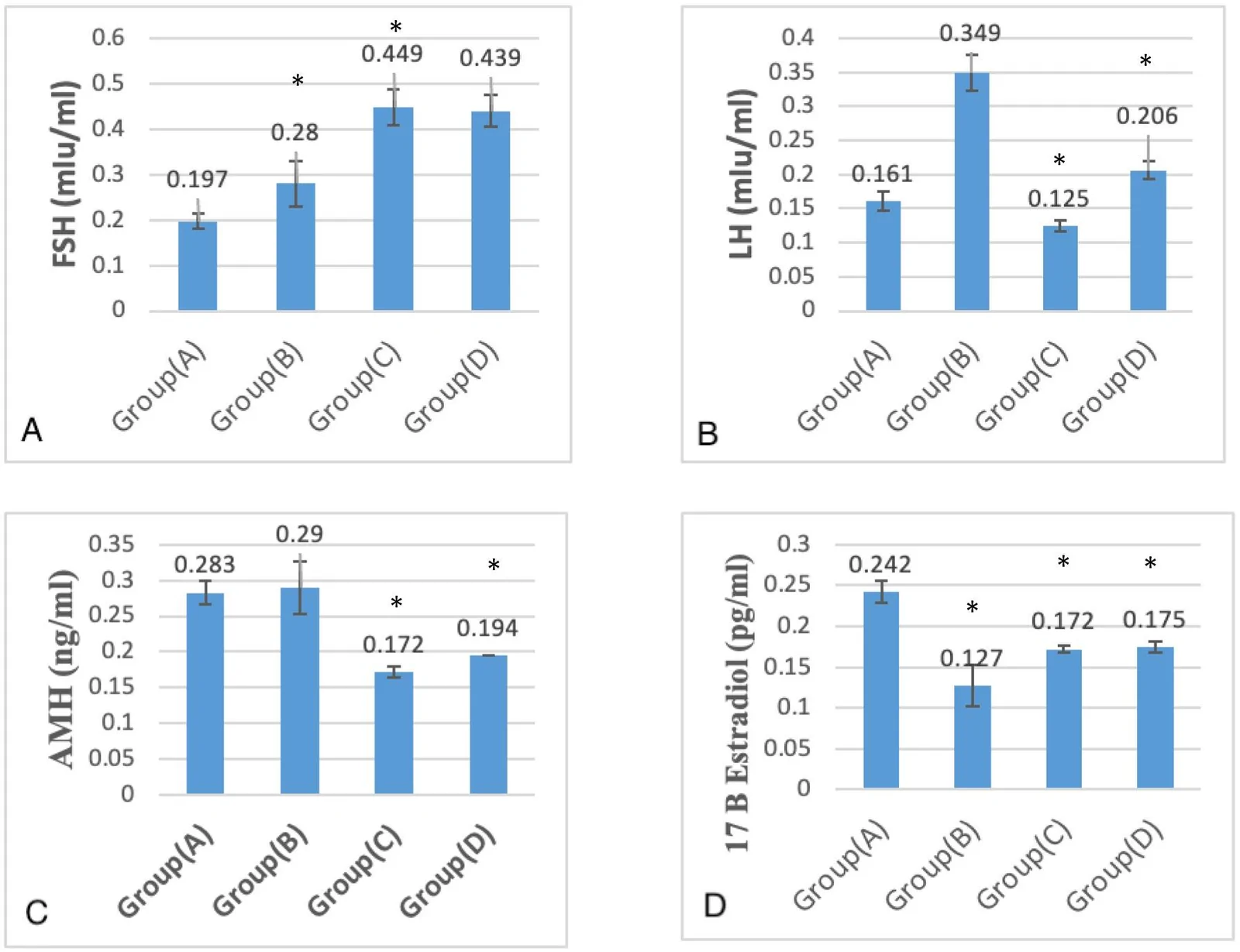
Effects of Protodioscin on Serum Reproductive Hormones.** A (FSH): **DOX treatment significantly elevated serum FSH levels by approximately 73.6% compared with control (*P* < 0.01), denoting follicular depletion and impaired negative feedback. Co-treatment with Protodioscin significantly decreased FSH by ~38% compared with DOX alone (*P* < 0.05), suggesting partial restoration of follicular reserve. **B (LH):** DOX exposure suppressed LH by 47.5% (*P* < 0.01), indicating hypothalamic–pituitary axis disruption. Protodioscin co-administration normalized LH levels (+81% vs. DOX, *P* < 0.05), reflecting recovery of gonadotropin regulation. **C (AMH):** Anti-Müllerian hormone (AMH) decreased by 46.7% after DOX exposure (*P* < 0.01), consistent with depletion of growing follicles. Co-treatment with Protodioscin increased AMH by ~75% relative to DOX (*P* < 0.05), restoring ovarian reserve. **D (E2):** Estradiol (E2) levels declined by 38.3% following DOX (*P* < 0.01), reflecting impaired steroidogenesis. Protodioscin significantly elevated E2 by ~54% compared with DOX (*P* < 0.05), indicating reactivation of granulosa cell function and aromatase activity.Data expressed as mean ± SD (n = 10)

###  Histopathological Observations

 Ovarian sections from control and protodioscin-treated groups exhibited normal histological architecture, including primary, secondary, and Graafian follicles, intact oocytes, and well-organized stromal tissue with corpus luteum structures (Figure S1, A–B). In contrast, DOX-treated rats showed marked histopathological damage, including follicular atresia, irregular oocyte morphology, stromal vacuolation, fibrosis, vascular congestion, hemorrhage, and disruption of the theca layer (Figure S1, C–E). Co-treatment with protodioscin markedly improved these alterations, with preservation of follicular architecture and reduced stromal damage. Several healthy follicles were observed, indicating partial structural recovery (Figure S1, F).

###  Immunohistochemical Analysis

 DOX administration significantly increased p53 expression while decreasing CDK4 and Survivin levels, indicating enhanced apoptotic activity and impaired cellular proliferation within ovarian tissue.

 Protodioscin co-treatment significantly attenuated these changes, reducing p53 immunoreactivity and increasing CDK4 and Survivin expression, suggesting a shift toward cell survival and proliferation (Table 5; Figures S2–S4).

**Table 5:** Semi-quantitative analysis of immunohistochemical markers in ovarian tissue

**Group**	**p53 (MOD)**	**CDK4 (MOD)**	**Survivin (MOD)**
Control	0.25 ± 0.03	0.70 ± 0.04	0.68 ± 0.05
Protodioscin	0.23 ± 0.02	0.74 ± 0.05*	0.72 ± 0.04*
DOX	0.85 ± 0.06**	0.33 ± 0.03**	0.30 ± 0.03**
DOX + Protodioscin	0.37 ± 0.04#	0.65 ± 0.04#	0.62 ± 0.05#

Data: mean optical density (MOD) ± SD. * *P* < 0.05 vs. Control; ** *P* < 0.01 vs. Control; # *P* < 0.05 vs. DOX.

###  Gene Expression Analysis

 Semi-quantitative RT-PCR analysis demonstrated that DOX significantly upregulated Bax expression and downregulated Bcl-2, resulting in an increased Bax/Bcl-2 ratio, indicative of activation of the intrinsic apoptotic pathway. Protodioscin co-treatment significantly modulated these alterations by reducing Bax expression and increasing Bcl-2 expression, thereby decreasing the Bax/Bcl-2 ratio (Table 6; Figures 3 and 4).

**Table 6:** Relative mRNA expression of Bax and Bcl-2 in ovarian tissues

**Group**	**Bax (fold change)**	**Bcl-2 (fold change)**	**Bax/Bcl-2 ratio**
Control	1.00 ± 0.07	1.00 ± 0.06	1.00
Protodioscin	0.95 ± 0.06	1.08 ± 0.05*	0.88
DOX	3.25 ± 0.11**	0.62 ± 0.08**	5.24
DOX + Protodioscin	1.28 ± 0.09#	1.15 ± 0.07#	1.11

Data: fold change relative to Control (mean ± SD). * *P* < 0.05 vs. Control; ** *P* < 0.01 vs. Control; # *P* < 0.05 vs. DOX.

**Figure 3:**
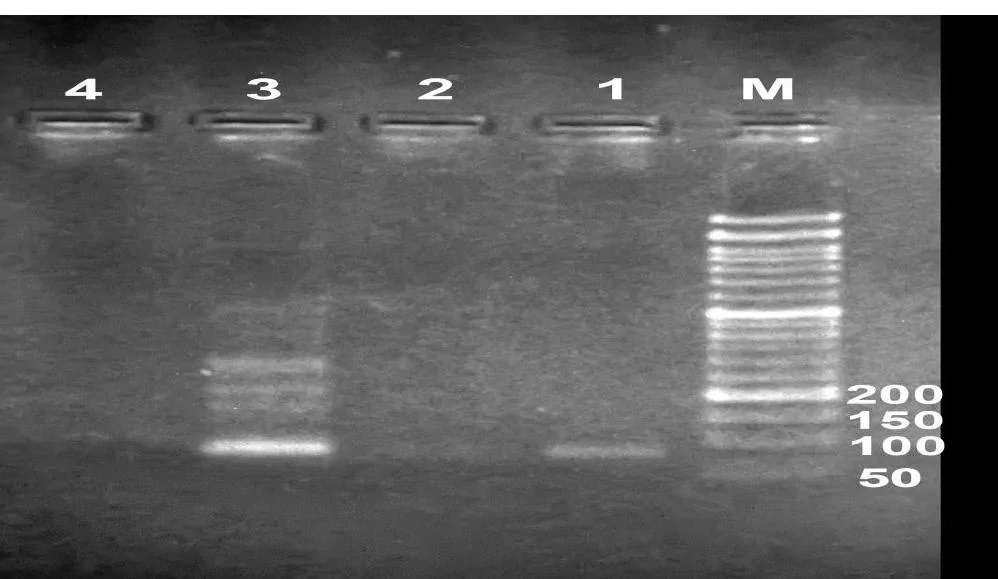
Relative mRNA Expression of Bax in Ovarian Tissue. Representative agarose gel bands with densitometric analysis normalized to β-actin. DOX exposure induced a 3.25-fold upregulation of Bax mRNA (*P* < 0.01), confirming activation of the mitochondrial apoptotic cascade. Co-treatment with Protodioscin significantly downregulated Bax by ~60% compared to DOX (*P* < 0.05), indicating effective suppression of intrinsic apoptotic pathways and mitochondrial protection

**Figure 4:**
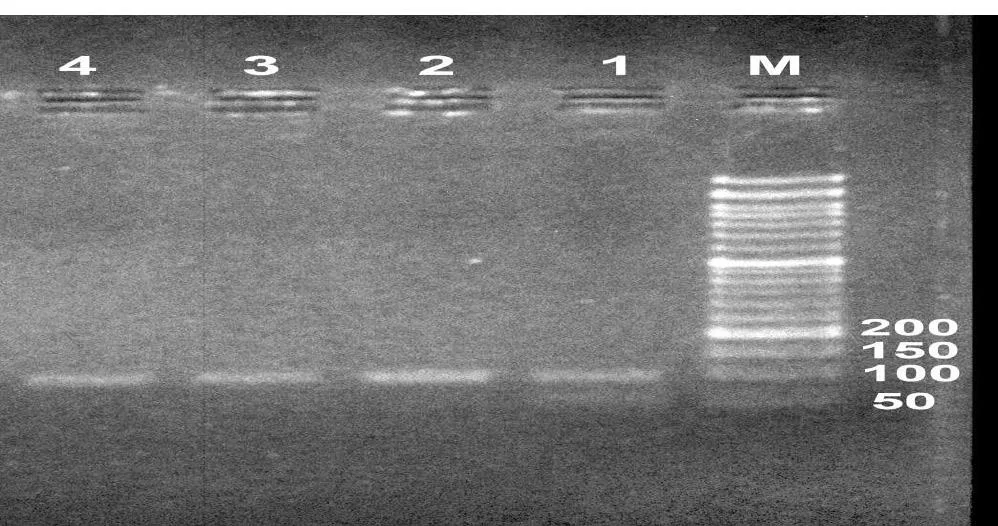
Relative mRNA Expression of Bcl-2 in Ovarian Tissue.Representative gel electrophoresis image showing Bcl-2 mRNA normalized to β-actin. DOX markedly suppressed Bcl-2 expression by ~38% compared to control (*P* < 0.01), confirming loss of anti-apoptotic signaling. Protodioscin alone caused a slight upregulation (+8%), while co-treatment with DOX + Protodioscin elevated Bcl-2 by ~85% vs. DOX (*P* < 0.05), restoring a favorable Bax/Bcl-2 ratio and improving follicular cell survival

## Discussion

 This investigation provides evidence that protodioscin, administered at 10 mg/kg daily for 14 days, exerts a protective effect against doxorubicin (DOX)-induced ovarian injury in female Wistar rats through modulation of hormonal and apoptotic signaling pathways.

 The present findings, supported by biochemical, histological, immunohistochemical, and molecular analyses, suggest that protodioscin contributes to the preservation of ovarian structure and function, potentially via regulation of hypothalamic–pituitary–gonadal (HPG) axis activity and attenuation of p53-associated apoptotic signaling. The observed reductions in body, ovarian, and uterine weights following DOX administration are consistent with previously reported systemic and reproductive toxicity induced by anthracyclines. Mechanistically, DOX exerts cytotoxic effects through DNA intercalation and stabilization of the topoisomerase II–DNA cleavage complex, leading to DNA double-strand breaks and activation of the p53-mediated apoptotic pathway.^15^ Subsequent activation of p53 promotes transcription of pro-apoptotic genes such as Bax, facilitating mitochondrial outer membrane permeabilization and release of cytochrome c, which initiates the caspase cascade and contributes to granulosa cell apoptosis.^16^ Meanwhile, suppression of the anti-apoptotic protein Bcl-2 shifts the balance toward cell death, resulting in follicular atresia, stromal damage, and impaired ovarian function. These molecular changes are consistent with the increased Bax/Bcl-2 ratio and elevated p53 expression observed in the DOX group, supporting activation of the intrinsic apoptotic pathway and aligning with the histopathological findings.^17^

 In contrast, protodioscin partially reversed these alterations, as evidenced by improvements in organ weights, hormonal profiles, and tissue morphology. The observed normalization of serum gonadotropins and estradiol levels suggests a partial restoration of HPG axis function; however, the absence of estrous cycle synchronization limits the interpretation of these hormonal changes. The steroid-like structure of protodioscin, classified as a furostanol saponin, may contribute to its biological activity by interacting with steroid-responsive pathways and enhancing ovarian steroidogenesis. This may explain the observed increase in estradiol levels and the associated feedback inhibition of FSH secretion, indicating partial restoration of endocrine homeostasis.^18^ At the molecular level, protodioscin attenuated the DOX-induced apoptotic response by downregulating p53 and Bax while upregulating Bcl-2, suggesting a shift toward cell survival. In addition, the restoration of CDK4 and Survivin expression indicates improved cellular proliferation and reduced apoptosis. CDK4 plays a critical role in granulosa cell cycle progression and follicular development, and its downregulation following DOX exposure has been associated with impaired folliculogenesis.^19,20^

 Protodioscin’s ability to restore CDK4 expression may contribute to follicular regeneration and improved ovarian morphology. Similarly, Survivin, a member of the inhibitor of apoptosis protein (IAP) family, inhibits caspase activation and promotes cell survival. The recovery of Survivin expression following protodioscin treatment suggests a reduction in apoptotic signaling and enhanced cellular resilience.^21^

 Collectively, the observed molecular changes reduced p53 and Bax levels alongside increased Bcl-2, CDK4, and Survivin expression indicate a restoration of mitochondrial integrity and a shift from apoptosis toward cell survival. These findings support the cytoprotective role of protodioscin in ovarian tissue.^22,23^ Additionally, protodioscin has been previously reported to possess antioxidant and anti-inflammatory properties, which may contribute to its protective effects by reducing oxidative stress and stabilizing mitochondrial function. Its ability to modulate intracellular signaling pathways may further explain its protective role against DOX-induced cellular damage.^24^ From a broader perspective, the observed modulation of apoptotic and proliferative pathways suggests that protodioscin may exert cytoprotective effects beyond the ovary. Dysregulation of similar pathways has been implicated in chemotherapy-induced cardiotoxicity, pancreatic β-cell apoptosis, hepatic injury, and neurodegenerative disorders. However, extrapolation of these findings to other tissues should be interpreted with caution, and further studies are required to confirm systemic effects.

 It is important to acknowledge certain limitations in this study. The lack of estrous cycle control represents a potential confounding factor in hormonal and histological assessments. Additionally, the use of a single-dose DOX model may not fully reflect the chronic and cumulative toxicity observed in clinical settings. Furthermore, the absence of direct mechanistic assays (e.g., oxidative stress markers or signaling pathway inhibition) limits definitive conclusions regarding the precise pathways involved. In conclusion, protodioscin demonstrated a protective effect against DOX-induced ovarian damage, primarily through modulation of apoptotic pathways and partial restoration of endocrine function. These findings support its potential as a candidate for further investigation as a fertility-preserving adjunct during chemotherapy. However, additional preclinical and clinical studies are required to validate these effects and clarify the underlying mechanisms.

## Conclusion

 Protodioscin attenuated doxorubicin-induced ovarian injury in this experimental model, as evidenced by improvements in hormonal profiles, histological structure, and apoptosis-related markers. The compound modulated the expression of p53, Bax, and Bcl-2, along with CDK4 and Survivin, suggesting a role in regulating cell survival pathways. However, due to limitations including the absence of estrous cycle control and the use of an acute DOX model, these findings should be interpreted with caution. Further studies are necessary to validate these results and determine whether the observed effects are ovary-specific or reflect broader systemic protection.

## Competing Interests

 The authors declare that they have no known competing financial interests or personal relationships that could have appeared to influence the work reported in this paper.

## Ethical Approval

 The experimental protocol was reviewed and approved by the Mansoura University Faculty of Science Ethics Committee (Approval No. Sci-Z-P-2023-123).

## Supplementary File

Supplementary file contains Figure S1-S4.

## References

[1] Bhadran A, Polara H, Babanyinah GK, Baburaj S, Stefan MC (2025). Advances in doxorubicin chemotherapy: emerging polymeric nanocarriers for drug loading and delivery. Cancers (Basel).

[2] Wang Y, Liu M, Johnson SB, Yuan G, Arriba AK, Zubizarreta ME (2019). Doxorubicin obliterates mouse ovarian reserve through both primordial follicle atresia and overactivation. Toxicol Appl Pharmacol.

[3] Ben-Aharon I, Bar-Joseph H, Tzarfaty G, Kuchinsky L, Rizel S, Stemmer SM (2010). Doxorubicin-induced ovarian toxicity. Reprod Biol Endocrinol.

[4] Jankowska K (2017). Premature ovarian failure. Prz Menopauzalny.

[5] Kciuk M, Gielecińska A, Mujwar S, Kołat D, Kałuzińska-Kołat Ż, Celik I (2023). Doxorubicin-an agent with multiple mechanisms of anticancer activity. Cells.

[6] Hao Q, Chen J, Lu H, Zhou X (2023). The ARTS of p53-dependent mitochondrial apoptosis. J Mol Cell Biol.

[7] Qian S, Wei Z, Yang W, Huang J, Yang Y, Wang J (2022). The role of Bcl-2 family proteins in regulating apoptosis and cancer therapy. Front Oncol.

[8] Shen YH, Peng S, Zhu T, Shen MJ (2025). Mechanisms of granulosa cell programmed cell death and follicular atresia in polycystic ovary syndrome. Physiol Res.

[9] Clayton ZS, Brunt VE, Hutton DA, VanDongen NS, D’Alessandro A, Reisz JA (2020). Doxorubicin-induced oxidative stress and endothelial dysfunction in conduit arteries is prevented by mitochondrial-specific antioxidant treatment. JACC CardioOncol.

[10] Jaiyeoba-Ojigho JE, Asiwe JN, Chimezie J, Abe BO, Ataikiru OM, Enaohwo TM (2025). Doxorubicin-mediated testicular toxicity is associated with dysregulated mTOR/Beclin-1 pathways, oxidative stress, inflammation and apoptosis in Wistar rats: preventive role of lutein. Lab Anim Res.

[11] Blumenfeld Z (2019). Fertility preservation using GnRH agonists: rationale, possible mechanisms, and explanation of controversy. Clin Med Insights Reprod Health.

[12] Wang ZF, Wang BB, Zhao Y, Wang FX, Sun Y, Guo RJ (2016). Furostanol and spirostanol saponins from Tribulus terrestris. Molecules.

[13] Al Zarzour RH, Kamarulzaman EE, Saqallah FG, Zakaria F, Asif M, Abdul Razak KN (2022). Medicinal plants’ proposed nanocomposites for the management of endocrine disorders. Heliyon.

[14] Pratt MA, Niu MY (2003). Bcl-2 controls caspase activation following a p53-dependent cyclin D1-induced death signal. J Biol Chem.

[15] Yang F, Teves SS, Kemp CJ, Henikoff S (2014). Doxorubicin, DNA torsion, and chromatin dynamics. Biochim Biophys Acta.

[16] Li M (2021). The role of P53 up-regulated modulator of apoptosis (PUMA) in ovarian development, cardiovascular and neurodegenerative diseases. Apoptosis.

[17] Wang XL, Wu Y, Tan LB, Tian Z, Liu JH, Zhu DS (2012). Follicle-stimulating hormone regulates pro-apoptotic protein Bcl-2-interacting mediator of cell death-extra long (BimEL)-induced porcine granulosa cell apoptosis. J Biol Chem.

[18] Acevedo-Rodriguez A, Kauffman AS, Cherrington BD, Borges CS, Roepke TA, Laconi M (2018). Emerging insights into hypothalamic-pituitary-gonadal axis regulation and interaction with stress signalling. J Neuroendocrinol.

[19] Wei H, Wang H, Wang G, Qu L, Jiang L, Dai S (2023). Structures of p53/Bcl-2 complex suggest a mechanism for p53 to antagonize Bcl-2 activity. Nat Commun.

[20] Tarasewicz E, Hamdan R, Straehla J, Hardy A, Nunez O, Zelivianski S (2014). CDK4 inhibition and doxorubicin mediate breast cancer cell apoptosis through Smad3 and survivin. Cancer Biol Ther.

[21] Tsutsui T, Hesabi B, Moons DS, Pandolfi PP, Hansel KS, Koff A (1999). Targeted disruption of CDK4 delays cell cycle entry with enhanced p27(Kip1) activity. Mol Cell Biol.

[22] Chen X, Duan N, Zhang C, Zhang W (2016). Survivin and tumorigenesis: molecular mechanisms and therapeutic strategies. J Cancer.

[23] Suroto H, Asriel A, De Vega B, Samijo SK (2021). Early and late apoptosis protein expression (Bcl-2, BAX and p53) in traumatic brachial plexus injury. J Musculoskelet Neuronal Interact.

[24] Yan F, Zhao Q, Li Y, Zheng Z, Kong X, Shu C (2022). The role of oxidative stress in ovarian aging: a review. J Ovarian Res.

