# Supplementary material for: Protodioscin Restores Ovarian Function and Suppresses p53–Bax/Bcl-2–Mediated Apoptosis in a Doxorubicin-Induced Model of Ovarian Toxicity
Source: Adv Pharm Bull. 2026 Jul 11;16(3):647–54. doi: 10.34172/apb.46593 (PMC13745742; doi:10.34172/apb.46593)
Supplement: Supplementary file 1 [file apb-16-647-s001.pdf]

Adv Pharm Bull 2026;16(3):647-S1
doi:10.34172/apb.46593
https://apb.tbzmed.ac.ir

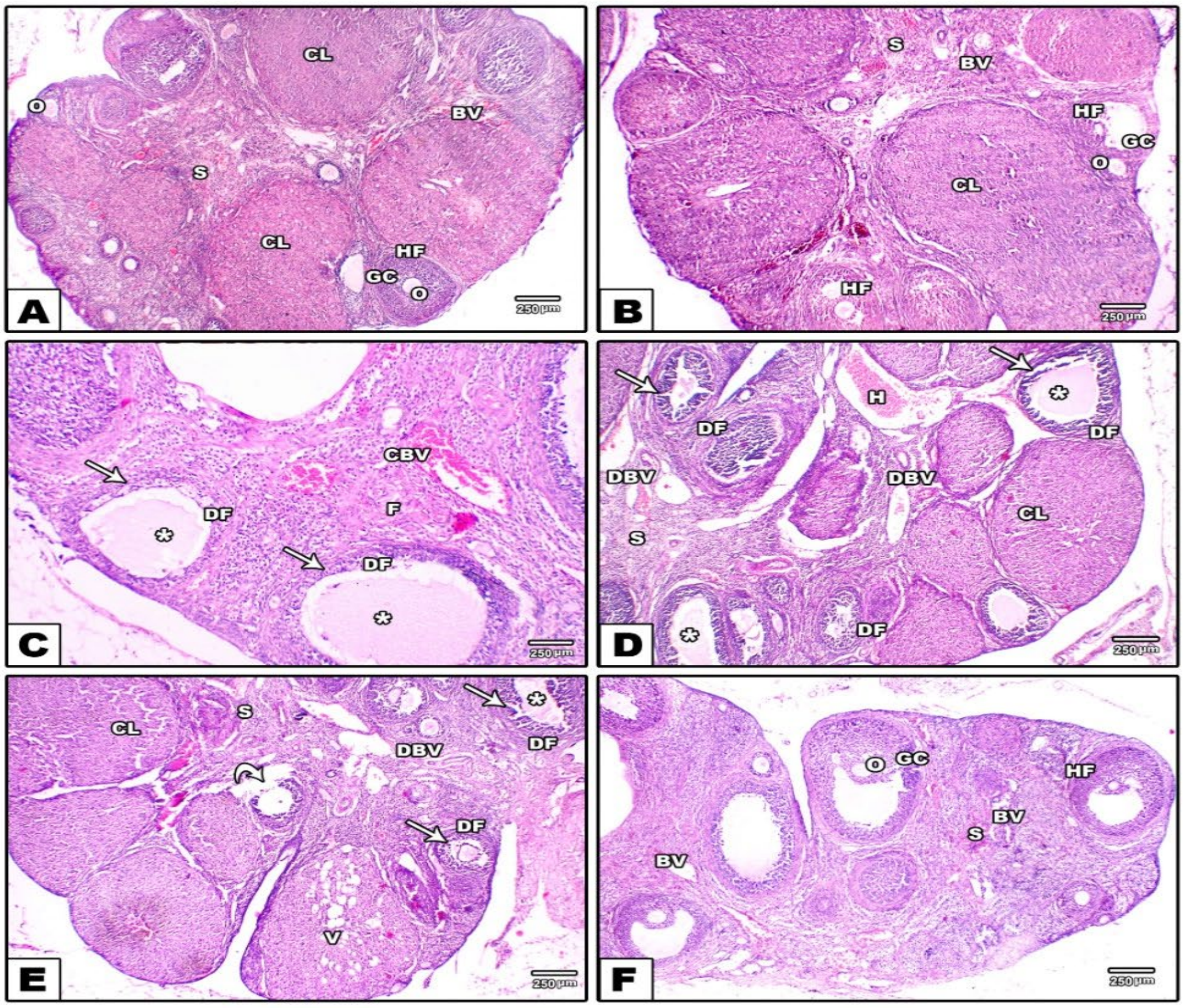

Figure S1: Histopathological Evaluation of Ovarian Sections (H&E, Bar = 5 µm)**. A (Control):** Ovarian section displays normal histoarchitecture with well-developed primary, secondary, and Graafian follicles. Oocytes exhibit clear nuclei, intact zona pellucida, and well-organized granulosa and theca layers. The stroma appears compact with abundant healthy blood vessels and active corpus luteum. **B (Protodioscin):** Tissue architecture remains comparable to control, with intact follicles and corpus luteum, indicating no histopathological alterations or cytotoxicity from Protodioscin. **C (DOX):** Sections reveal extensive degenerative and atretic changes. Follicles appear shrunken and irregular with disrupted granulosa layers, pyknosis of oocyte nuclei, cytoplasmic vacuolization, stromal fibrosis, hemorrhage, and congested or necrotic blood vessels. The follicular wall exhibits thinning and disorganization, confirming severe DOX-induced oxidative and apoptotic injury. **F (DOX + Protodioscin):** Co-treatment substantially reversed DOX-induced pathology. Ovarian follicles displayed restored morphology with normal granulosa layering, viable oocytes, revascularized stroma, and multiple active corpus lutea. These findings denote effective tissue regeneration and anti-apoptotic protection by Protodioscin.

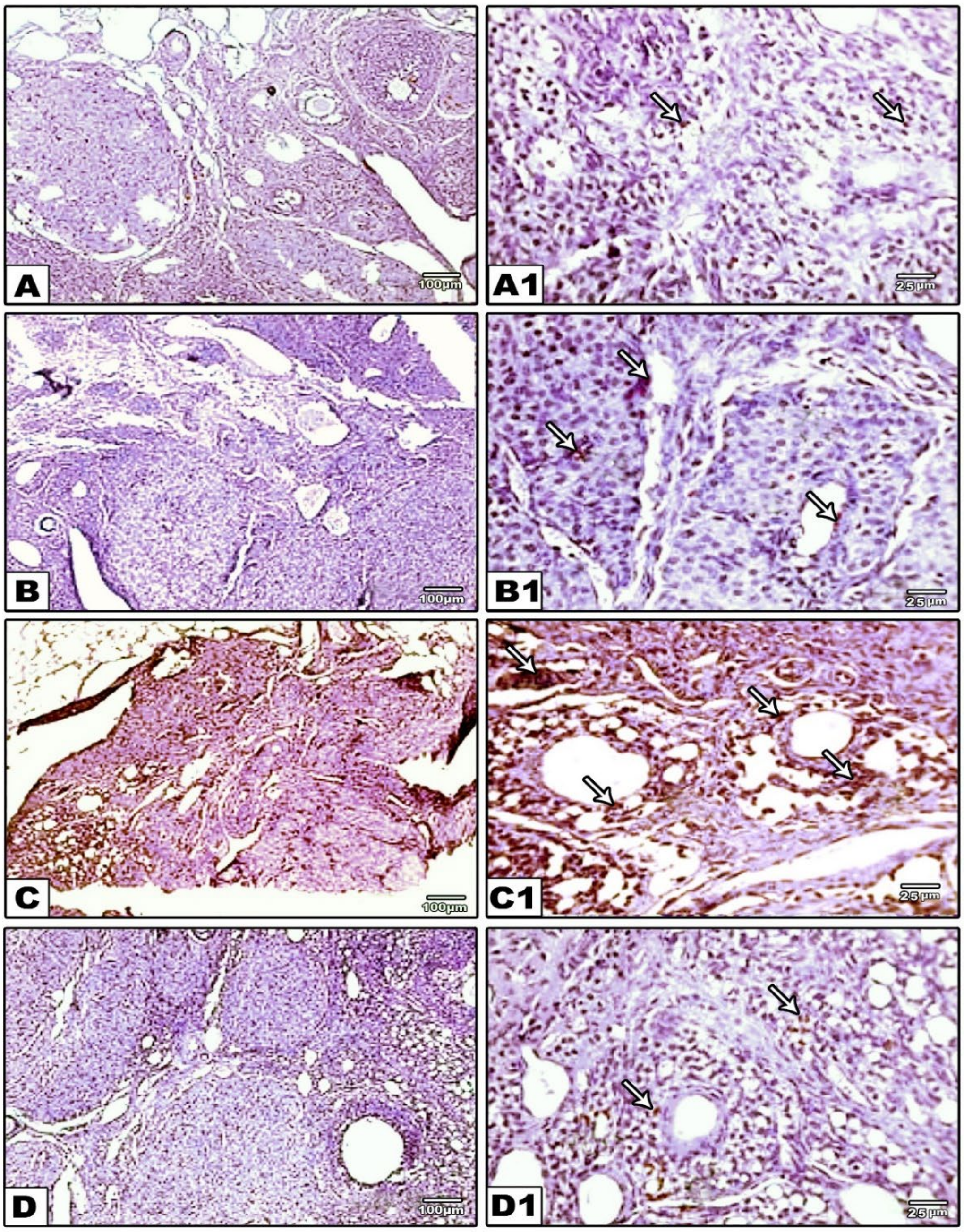

Figure S2: Immunohistochemical Detection of p53 in Ovarian Tissue (×400). **A (Control):** Weak nuclear p53 immunostaining in scattered granulosa cells, representing basal apoptotic turnover. **B (Protodioscin):** Similar weak-to-moderate nuclear reactivity, confirming the absence of apoptotic stimulation. **C (DOX):** Strong, diffuse p53 nuclear positivity, particularly in atretic follicles and degenerated stromal regions. Quantitatively, p53 expression increased ~3.4-fold compared with control ($P < 0.01$), consistent with DOX-induced DNA damage and activation of intrinsic apoptosis. **D (DOX + Protodioscin):** Co-treatment markedly reduced p53 immunoreactivity (~56% decrease vs. DOX, $P < 0.05$), restoring near-control levels, indicating Protodioscin-mediated inhibition of apoptotic signaling and genomic stress.

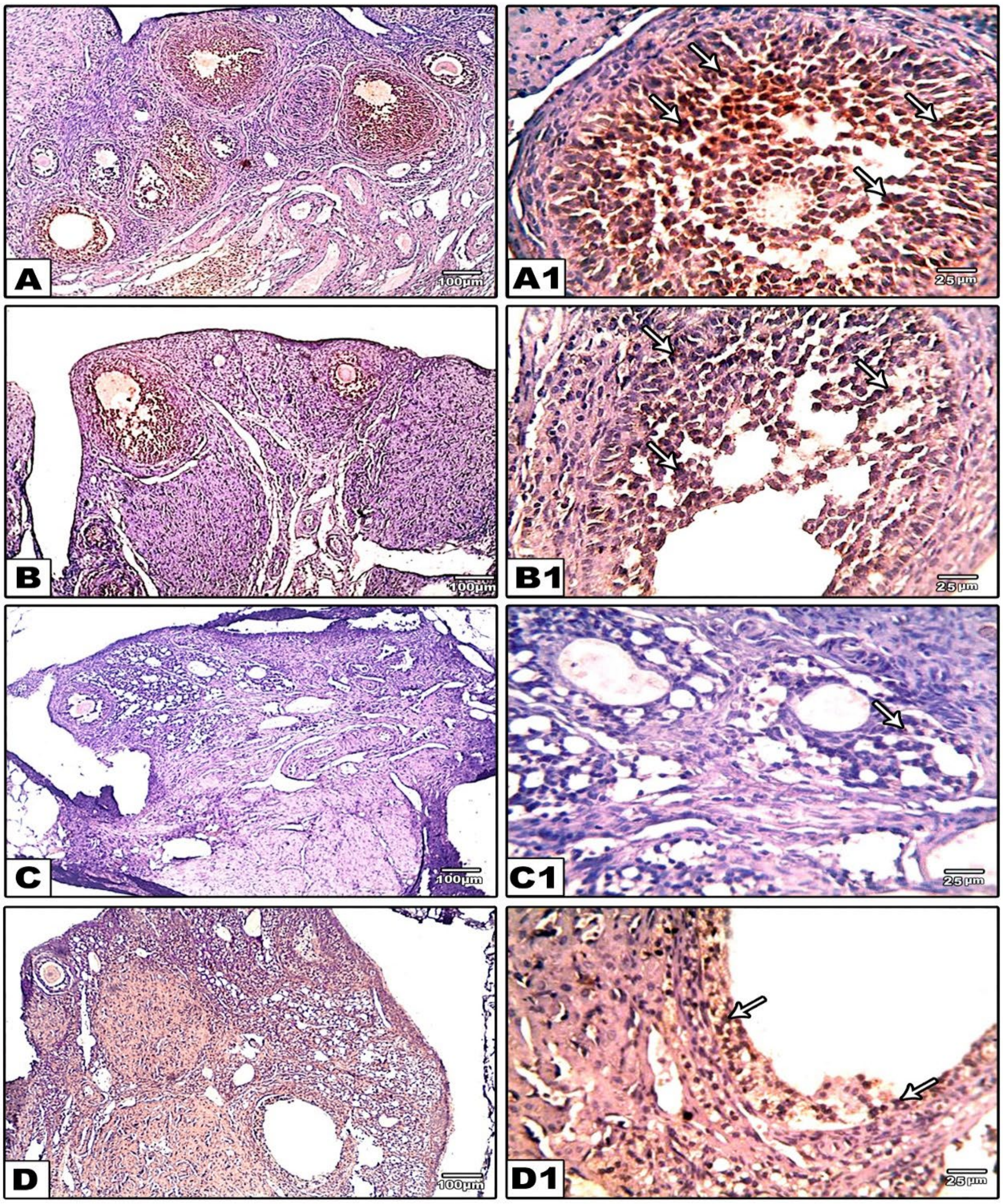

Figure S3: Immunohistochemical Detection of CDK4 (×400). **A (Control):** Strong nuclear CDK4 expression in granulosa, theca, and stromal cells, reflecting active cell cycle progression and follicular proliferation. **B (Protodioscin):** Comparable strong CDK4 staining pattern to control, indicating sustained mitotic activity and normal folliculogenesis. **C (DOX):** Weak and patchy CDK4 expression, primarily confined to residual stromal cells. Quantitatively, a ~53% reduction vs. control ($P < 0.01$) was observed, confirming DOX-induced G1/S phase arrest and impaired proliferative capacity. **D (DOX + Protodioscin):** Co-treatment restored CDK4 expression to nearly double the DOX group level ($P < 0.05$), reactivating proliferation and follicular regeneration, suggesting direct modulation of cell cycle checkpoints by Protodioscin.

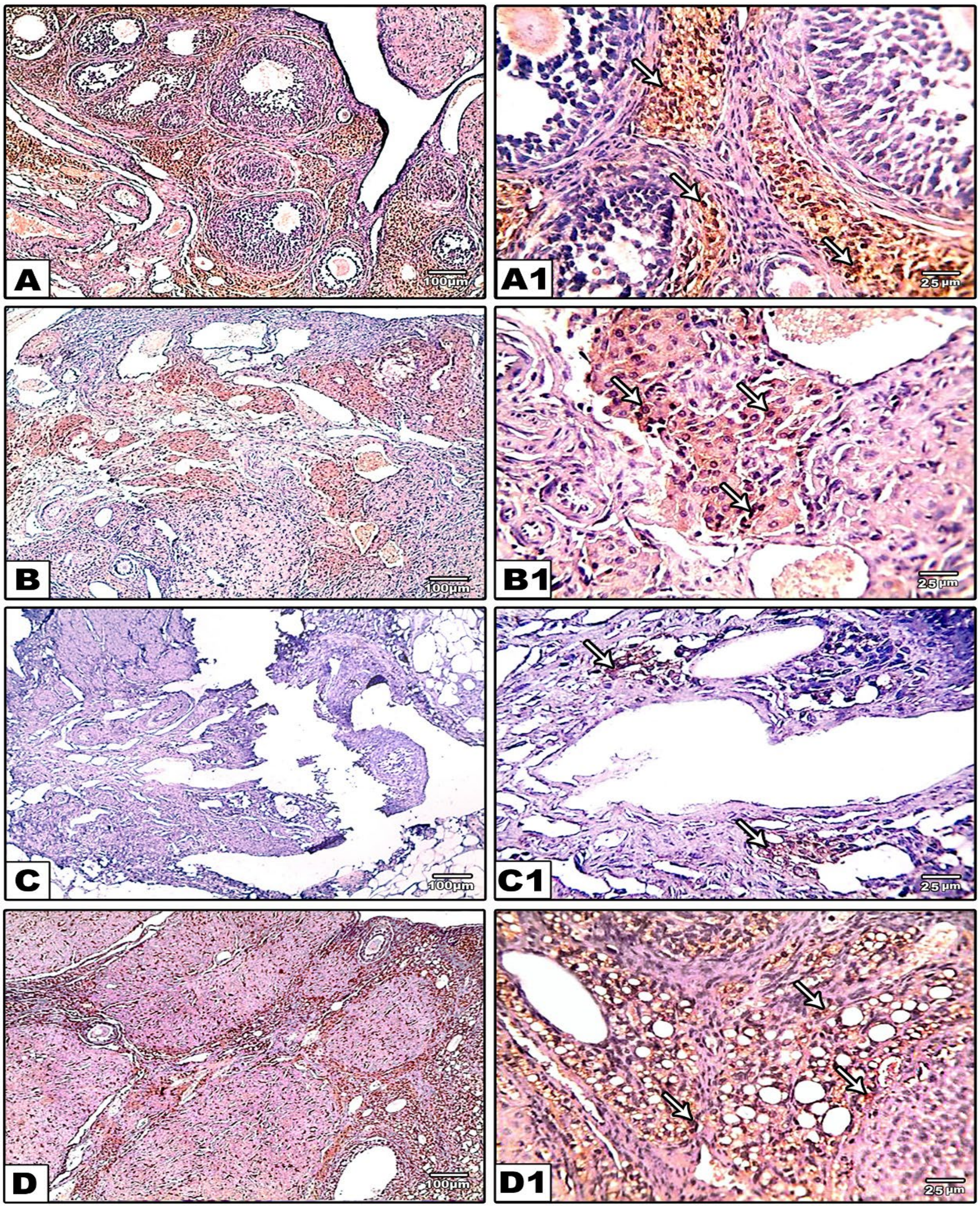

Figure S4: Immunohistochemical Detection of Survivin (×400)**. A (Control):** Strong cytoplasmic Survivin immunoreactivity within granulosa and luteal cells, indicating active anti-apoptotic signaling and normal follicular viability. **B (Protodioscin):** Survivin expression remains high, similar to control, confirming absence of pro-apoptotic stress. **C (DOX):** Weak and sparse Survivin staining, reduced by ~56% vs. control ($P < 0.01$), corresponding with elevated apoptosis and follicular atresia. **D (DOX + Protodioscin):** Robust Survivin re-expression (~2.1-fold vs. DOX, $P < 0.05$) in granulosa and stromal cells, reflecting restoration of anti-apoptotic defense and enhanced cellular survival mechanisms.
